# Supplementary figures and images for: Comparison of an Artificial Neural Network and a Response Surface Model during the Extraction of Selenium-Containing Protein from Selenium-Enriched Brassica napus L
Source: Foods. 2022 Nov 27;11(23):3823. doi: 10.3390/foods11233823 (PMC9740868; doi:10.3390/foods11233823)

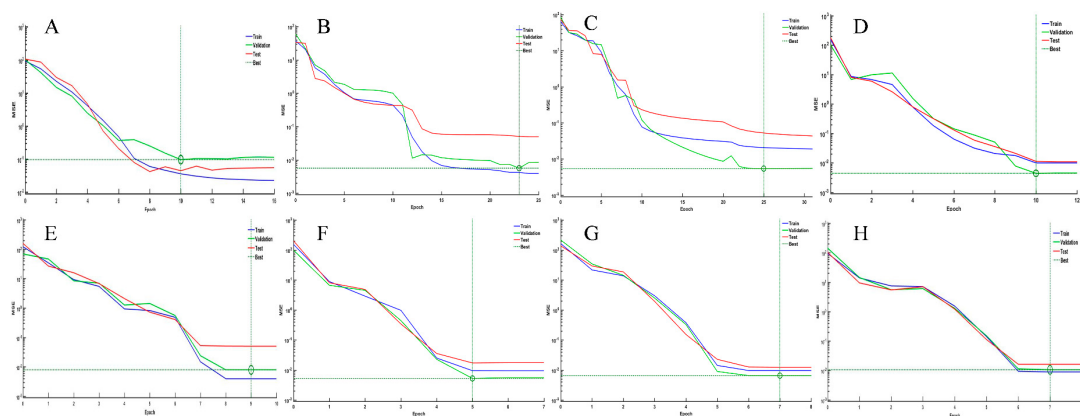

**Figure S1.** MSE of BP neural networks comprising 4-11 nodes (A-H) in the single hidden layer

Supplement: Supplementary file 1 [file foods-11-03823-s001.zip › foods-1971860-supplementary.pdf]
